# Functional capacity and heart rate response: associations with nocturnal hypertension

**DOI:** 10.1186/s12872-015-0064-7

**Published:** 2015-07-22

**Authors:** Paul Ritvo, Leslie E. Stefanyk, Saam Azargive, Slobodan Stojanovic, Faye Stollon, Juda Habot, Yaariv Khaykin, Terry Fair, Meysam Pirbaglou

**Affiliations:** School of Kinesiology and Health Science, York University, Toronto, ON Canada; Department of Psychology, York University, Toronto, ON Canada; Research, Prevention and Cancer Control, Cancer Care Ontario, Toronto, ON Canada; Department of Psychiatry, University of Toronto, Toronto, ON Canada; Department of Community and Family Medicine, University of Toronto, Toronto, ON Canada; Division of Cardiology, Southlake Regional Health Centre, Newmarket, ON Canada; Faculty of Medicine, University of Toronto, Toronto, ON Canada

**Keywords:** Ambulatory blood pressure monitoring, Abnormal nocturnal blood pressure, Nocturnal non-dipping

## Abstract

**Background:**

Absences of normative, 10–20 % declines in blood pressure (BP) at night, termed nocturnal non-dipping, are linked to increased cardiovascular mortality risks. Current literature has linked these absences to psychological states, hormonal imbalance, and disorders involving hyper-arousal. This study focuses on evaluating associations between nocturnal non-dipping and indices of functional cardiac capacity and fitness.

**Methods:**

The current study was a cross-sectional evaluation of the associations between physical capacity variables e.g. Metabolic Equivalent (MET) and Maximum Heart Rate (MHR), Heart rate reserve (HRR), and degree of reduction in nocturnal systolic blood pressure (SBP) or diastolic blood pressure (DBP), also known as ‘dipping’. The study sample included 96 cardiac patient participants assessed for physical capacity and ambulatory blood pressure monitoring. In addition to evaluating differences between groups on nocturnal BP ‘dipping’, physical capacity, diagnoses, and medications, linear regression analyses were used to evaluate potential associations between nocturnal SBP and DBP ‘dipping’, and physical capacity indices.

**Results:**

45 males and 14 females or 61.5 % of 96 consented participants met criteria as non-dippers (<10 % drop in nocturnal BP). Although non-dippers were older (*p* = .01) and had a lower maximum heart rate during the Bruce stress test (*p* = .05), dipping was only significantly associated with Type 2 Diabetes co-morbidity and was not associated with type of medication. Within separate linear regression models controlling for participant sex, MHR (β = 0.26, *p* = .01, R^2^ = .06), HRR (β = 0. 19, *p* = .05, R^2^ = .05), and METs (β = 0.21, *p* = .04, R^2^ = .04) emerged as significant but small predictors of degree of nighttime SBP dipping. Similar relationships were not observed for DBP.

**Conclusions:**

Since the variables reflecting basic heart function and fitness (MHR and METs), did not account for appreciable variances in nighttime BP, nocturnal hypertension appears to be a complex, multi-faceted phenomena.

## Background

Blood pressure (BP) reductions of 10–20 % during sleep are essential for heart health [1–3]. When these reductions don’t occur, the resulting abnormal nocturnal blood pressure (ANBP) is associated with increased cardiovascular mortality risks [1–3]. Nighttime non-reducing or ‘non-dipping’ BP patterns (<10 % decline in night-time BP relative to daytime values) are associated with excess sympathetic activity [4], sleep-disordered breathing [5], increased vasoactive hormone secretion [6], impaired renal function [7, 8], target organ damage [9], and overall increased cardiovascular mortality risk which can be as much as 40 % higher than risks associated with normal nocturnal BP [1].

Recent studies have linked ANBP and decreased nighttime BP reductions to disorders involving hyperarousal and disrupted sleep (e.g. chronic insomnia). In these studies, non-dipping was associated with greater beta frequency in sleep EEG [10], and PTSD diagnosis, where non-dippers reported hyper-arousal symptoms, poor sleep quality, and longer sleep onset latency [11]. ANBP has also been associated with job strain [12, 13], depression [14], exhaustion [15], self reported stress and neighborhood (external) stressors [16, 17], highlighting its linkage with variations in physiological arousal.

Currently, ANBP diagnosis requires 24-h ambulatory blood pressure monitoring (ABPM), and despite epidemiological studies highlighting the importance of ANBP in improved hypertension management [3, 18, 19], the difficulty of conducting serial ABPM assessments has limited ABPM-guided ANBP treatment. Along these lines, an important question is the degree to which ANBP appears linked to irreversible deteriorations of the cardiovascular and autonomic nervous systems. If data do not suggest such a link, between nocturnal non-dipping and irreversible cardiovascular and autonomic deteriorations, there appears potential to reduce ANBP with a combination of medical and behavioural treatments.

While existing literature has focused on the link between hyper-arousal and ANBP [4, 10, 12], less attention has been directed to the potential associations between ANBP and fundamental indices of functional cardiac capacity and fitness (e.g. exercise capacity). Exercise capacity and the cardiovascular response to exercise are routinely assessed in cardiac rehabilitation settings for both diagnostic (e.g. identifying myocardial ischemia) and prognostic (e.g. risk stratification) purposes [20] because reduced exercise capacity [21–23] and sub-optimal heart rate response to exercise (e.g. inadequate increase in heart rate during exercise, abnormal heart rate recovery after exercise) [24, 25] indicate cardiac dysfunctions that strongly predict mortality and disease severity. Given the absence of evaluations of exercise capacity and fitness in relation to ANBP (and reduced dipping), our study objective was to assess associations between ABP-related values, most importantly ANBP, with indices of physical capacity (i.e. maximum Heart rate (MHR) and functional capacity expressed in metabolic equivalents (METS). In addition, to account for individual or sex differences in resting heart rate, we explored similar associations between ANBP and heart rate reserve (HRR), expressed as the difference between MHR and resting heart rate. Such findings can indicate whether ANBP is associated with fundamental and possibly irreversible cardiac deficits. Alternatively, modest associations might suggest potential ANBP modifiability through medical and behavioural treatment.

## Methods

### Study design and participants

The current study is a cross-sectional evaluation of associations between the extent of nocturnal dipping in blood pressure and physical capacity variables assessed through a standard cardiovascular stress test. Study participants were enrolled in the Cardiovascular Prevention and Rehabilitation Program at Southlake Regional Health Centre (Newmarket, ON). All individuals entering the program received information about the research study through an information sheet, and verbally through Rehabilitation Program staff. Individuals who expressed interest were directed to research staff who helped them complete an informed consent form for subsequent 24-h ambulatory blood pressure monitoring and to allow research staff to access their stress test results. A total of 96 individuals consented and took part in both the 24-h blood pressure monitoring and exercise capacity evaluation. The study was reviewed and approved by Human Participants Review Committee at York University in Toronto, Canada.

### Study parameters

The 24-h blood pressure (BP) assessment (Spacelabs Healthcare, Snoqualmie, WA) entailed a BP measurement every 20 min during waking periods and every hour during sleep. The less frequent hourly assessments (during sleep) were deliberately selected because more frequent assessments (during sleep) were perceived to likely be sleep disruptive. We included a 24-h assessment of systolic blood pressure (SBP), diastolic blood pressure (DBP), mean arterial pressure (MAP), pulse pressure (PP), and heart rate (HR). To allow a better understanding of blood pressure variation in relation to their day and night activities, participants kept logs of daily activities, along with times for initiating and waking up from sleep. Participants had previously self-disclosed their typical bedtime and sleep time (prior to the 24 h ABPM), and we used their disclosures to conclude the assessments per 20 min intervals, and initiate the assessments per 60 min intervals. If patient logs during the 24 h ABPM diverged from their prior self-report, we corrected the record so actual bedtime and sleep times were actually associated with nocturnal BP assessments.

The primary dependent measure was the percent nocturnal reduction in blood pressure in relation to both mean systolic blood pressure (SBP) and the diastolic blood pressure (DBP). This reduction value was calculated as [daytime SBP or DBP – nighttime SBP or DBP]/daytime SBP or DBP expressed as a percentage [26, 27]. Participants were classified as BP non-dippers if their nocturnal BP drop was less than 10 % and BP dippers if they showed equal or greater than 10 % drop in their nocturnal BP relative to daytime values. [26]. The stress test completed by each individual followed the Bruce or modified Bruce treadmill protocol [28] which are non-invasive measures of functional capacity and exercise tolerance in individuals with known or suspected cardiovascular disorders. Using these protocols, functional capacity is represented by the maximal workload attained and expressed in metabolic equivalents (MET). Other relevant performance variables included MHR achieved during the stress test protocol and test duration. Blood pressure and resting heart rate were taken before and at the termination of the stress test in accordance with the test protocol. Other study measures obtained on the day of the stress test included height, weight, Body Mass Index (BMI), and waist circumference (WC).

### Statistical analysis

Descriptive statistics on all demographic and clinical variables are presented in Table 1. Numeric variables are presented as means and standard deviation while categorical variables are presented in terms of frequency and percentage. We used independent samples *t*-test to evaluate potential differences between males and females and BP dippers and BP non-dippers in relevant demographic, clinical, body composition, fitness, and ambulatory blood pressure variables. Where applicable, we report t-tests with Welsh correction to account for unequal variances between groups. We used chi-square test for independence to explore associations between categorical study variables (e.g. sex or BP dipping status as categorical variables). Similarly, Pearson product–moment correlation coefficients are reported for continuous variables. Finally, associations between SBP and DBP dipping and functional capacity variables, such as metabolic equivalent (MET), MHR, or alternatively HRR were investigated within separate multiple linear regression models. In addition, we assessed possible interactions between these variables and sex within the regression models. All analyses were performed with SPSS v.19 (SPSS, IBM Corp.).

**Table 1:** Demographic and clinical characteristics according to SBP dipping status

Variables		Dippers (*n* = 37)	Non-dippers (*n* = 59)	*P*
Age (years)		60.6 (10.6)	65.9 (9.0)	.01
BMI (kg/m^2^)		29.1 (4.3)	30.0 (5.9)	.42
WC (cm)		99.4 (13.9)	104.1 (14.8)	.13
Resting Heart Rate (bpm)		69.0 (11.0)	67.3 (13.9)	.55
Heart Rate Reserve		61.7 (20.7)	54.01 (21.1)	.10
Maximum Heart Rate		130.3 (23.5)	121.3 (21.0)	.05
METs		7.8 (3.3)	6.7 (3.2)	.12
Additional Diagnosis				
	Cerebrovascular Accident	7.0 (18.9)	7.0 (11.8)	.34
	Congestive Heart Failure	3.0 (8.1)	2.0 (3.4)	.31
	Diabetes Mellitus	5.0 (13.5)	19.0 (32.3)	.04
	Pulmonary Disorder	4.0 (10.8)	5.0 (8.5)	.70
	Renal Failure	1.0 (2.7)	1.0 (1.7)	.73
Cardiovascular Medications				
	ACE Inhibitors	21.0 (56.8)	28.0 (47.5)	.37
	Angiotansin II Receptor Blockers	7.0 (18.9)	14.0 (23.7)	.57
	Beta Blockers	24 (64.9)	44.0 (74.6)	.30
	Calcium Channel Blockers	9.0 (24.3)	13.0 (22.0)	.79
	Diuretics	6.0 (16.2)	14.0 (23.7)	.37
	Platelet Aggregation Inhibitor	32.0 (86.5)	56.0 (94.9)	.14
24-h Ambulatory Blood Pressure (mmHg)				
	Daytime SBP	129.6 (11.2)	122.3 (12.8)	.006
	Nighttime SBP	110.1 (11.2)	117.4 (13.8)	.01
	Daytime DBP	77.4 (6.9)	74 (6.7)	.02
	Nightitime DBP	64.1 (6.8)	67.9 (7.1)	.01
	Daytime MAP	93.5 (10.5)	89.0 (13.8)	.09
	Nighttime MAP	80.9 (7.7)	85.1 (8.2)	.01
	Daytime PP	52.0 (9.2)	47.7 (11.7)	.06
	Nighttime PP	46.5 (8.6)	49.6 (11.2)	.16
24-h Heart Rate (bpm)				
	Daytime HR	72.7 (11.5)	70.0 (11.3)	.27
	Nighttime HR	65.1 (9.4)	64.1 (11.4)	.65

All values are presented as means (standard deviations) for numeric and frequency (percentage) for categorical variables. *BMI* body mass index, *WC* Waist circumference, *METS* metabolic equivalent. *SBP* systolic blood pressure, *DBP* diastolic blood pressure, *PP* pulse pressure, *MAP* mean arterial pressure, *HR* heart rate

## Results

Participants included 96 (69 males and 27 females) individuals with complete 24-h ABPM and functional capacity measurements. On average, 41.1 successful readings were taken during the daytime and 8.6 readings during nighttime. Using the commonly applied abnormal nocturnal BP (non-dipping) criteria of < 10 % drop in nocturnal blood pressure relative to daytime values, 45 males and 14 females or 61.5 % of participants met criteria for abnormal nocturnal BP (non-dippers). Compared to dippers (≥10 % nocturnal dipping), non-dippers were older (*p* = .01) and achieved a lower maximum heart rate during the Bruce stress test (*p* = .05). Non-dippers had also lower mean daytime SBP (*p* = .006) and DBP (*p* = .02) and higher mean nocturnal SBP (*p* = .01) and DBP (*p* = .01) values. With regards to diagnostic and medication variables, as presented in Table 1, except for type 2 Diabetes co-morbidity (*χ*^2^ = 4.23, *p* = .04), there were no associations between dipping status and other treatment-related variables, including medical diagnoses, cardiac procedure-treatment (including treatment for sleep apnea with chronic positive airway pressure) and, notably types of blood pressure medications.

Table 2 presents correlations between mean values in study variables and the extent of nocturnal SBP and DBP dipping as continuous variables according to participant sex. Among males, specifically, age (r = −.23, *p* = .05) along with MHR (r = .27, *p* = .02) had a significant correlation with the degree of nocturnal dipping. In addition, within a linear regression model that included participant sex, with males as the reference group (see Table 3), MHR (β = 0.26, *p* = .01) emerged as a significant predictor of the degree of nighttime SBP dipping in a model that, altogether, accounted for a relatively modest amount of variance (Adjusted R^2^ = .06). To further address individual and sex-related differences in resting heart rate, we ran a separate model with Heart Rate Reserve (HRR) as predictor variables. In this model, HRR was only also associated, albeit modestly, with the degree of SBP dipping (β = 0. 19, *p* = .05, R^2^ = .05). Along similar lines, functional capacity expressed in METS only marginally correlated with the extent of nocturnal dipping (r = .22, *p* = .07 for men and r = .18, *p* = .36 for women), and within a regression model that included participant sex (with males as the reference group), was a significant but small predictor of the degree of nighttime SBP dipping (β = 0.21, *p* = .04). Altogether, the overall model accounted for only 4 % of variance in nighttime SBP dipping (Adjusted R^2^ = .04). Similar regression models ran for diastolic blood pressure dipping (DBP) as outcome variable did not reach statistical significance.

**Table 2:** Pearson correlations between study variables and SBP and DBP dipping status according to sex

Variables	Males				Females			
	SBP Dipping	*P*	DBP Dipping	*P*	SBP Dipping	*P*	DBP Dipping	*P*
Age (years)	-.23	.05	-.23	.05	-.30	.12	-.16	.42
BMI (kg/m^2^)	-.13	.28	-.16	.18	-.23	.23	-.23	.24
WC (cm)	-.14	.23	-.16	.18	-.35	.07	-.32	.11
Resting Heart Rate (bpm)	.12	.33	.12	.29	.18	.37	.23	.24
Heart Rate Reserve	.21	.08	.17	.15	.18	.36	-.009	.96
Maximum Heart Rate	.27	.02	.24	.04	.25	.21	.12	.53
METs	.22	.07	.19	.11	.18	.36	.05	.77
24-h Ambulatory Blood Pressure (mmHg)								
Daytime SBP	.28	.01	.27	.02	-.10	.60	-.17	.39
Nighttime SBP	-.32	.007	-.18	.12	-.71	.0001	-.66	.0001
Daytime DBP	.28	.01	.30	.01	.14	.48	.10	.59
Nighttime DBP	-.30	.01	-.43	.0001	-.58	.002	-.73	.0001
Daytime PP	.17	.16	.15	.22	-.18	.35	-.23	.24
Nighttime PP	-.21	.07	.04	.70	-.55	.003	-.33	.08
Daytime MAP	.16	.18	.23	.05	-.03	.87	.06	.75
Nighttime MAP	-.32	.007	-.30	.01	-.67	.0001	-.74	.0001
24-h Heart Rate (bpm)								
Daytime HR	.004	.97	.10	.39	.32	.10	.39	.04
Nighttime HR	-.07	.54	-.01	.92	.09	.63	.09	.64

*BMI* body mass index, *WC* Waist circumference, *METS* metabolic equivalent, *SBP* systolic blood pressure, *DBP*, diastolic blood pressure, *PP* pulse pressure, *MAP* mean arterial pressure, *HR* heart rate

**Table 3:** Summary of linear regression analyses on the influence of functional capacity variables on SBP dipping

	Variables	b (SE)	β	*P*	Adj. R^2^
Model 1					.06
	Sex (male)	1.47 (1.41)	.10	.30	
	Maximum Heart Rate	.76 (.03)	.26	.01	
Model 2					.05
	Sex (male)	1.96 (1.43)	.13	.17	
	Heart Rate Reserve	.06 (.03)	.19	.05	
Model 3					.04
	Sex (male)	3.07 (1.47)	.21	.04	
	METS	.42 (.20)	.21	.04	

## Discussion

In this study our objective was to understand the relationship between dipping status in systolic blood pressure (SBP) and functional capacity indices, notably the maximum heart rates (MHRs) and metabolic equivalents (METs) achieved during standard stress tests with patients enrolled in a cardiac rehabilitation program. In addition, to control for sex or individual differences in MHR, we used HRR to account for individual or sex-related differences in resting heart rate. The control of nocturnal BP is an important cardiac risk target. Although current data are insufficient to demarcate the pathways of effective reduction, our results indicated cardiac performance (reflected by the MHR) and functional capacity (reflected in METs) during stress testing, was significantly but modestly associated with systolic blood pressure dipping.

Since the variables measured, reflecting basic heart function and fitness, do not account for an appreciable variance in nighttime BP, the nocturnal hypertension phenomena appears to be multi-faceted and complex. This raises the question of the impact of factors relating more broadly to autonomic sympathetic hyperarousal, both behavioural and physiological, and possibly modifiable via medication and behavior modification. Along these lines, the interventions thus far most frequently applied employ exercise and use of continuous positive airway pressure (CPAP) in managing nocturnal hypertension [29]. Wuerzner et al. [30] suggest higher physical activity levels were significantly related to reduced nocturnal hypertension levels, but when physical training intervention was specifically assessed, [31] aerobic training had no effect on most subjects (*N* = 14), and worsened the status of more subjects than it improved (5 subjects who were dippers became non-dippers and 4 subjects who were non-dippers became dippers). These results again suggest the the complexity of nocturnal BP which may not be linearly influenced by exercise training. Training may require adjustments not typically undertaken in exercise programs targeted at generic fitness. When non-dippers are found to have Obstructive Sleep Apnea (OSA), the use of continuous positive airway pressure (CPAP) has been identified, in some cases, as effective [29]. In our data, only 3 patients had been diagnosed and treated for OSA. Of the 3 patients, 1 had a nocturnal hypertension problem while 2 others did not. These results, in the patients assessed, do not indicate that OSA diagnosis and CPAP are a definitive treatment for nocturnal hypertension and the changes observed did not impact the model calculations derived.

In past research, multiple studies implicate disrupted sleep and autonomic hyperarousal as important factors that must be considered in the treatment of nocturnal hypertension. Population-based studies reveal an increased prevalence of sleep disorder likely attributable to increased daily stress. For example, the Sao Paulo Sleep Study [32] revealed a 32 % prevalence for objective insomnia (*N* = 1042), and the New Zealand blood donor study (*N* = 22,389) reported a 45 % prevalence of insomnia symptoms on at least one occasion per week [33]. A Canadian study (*N* = 2000) identified 40 % of individuals reporting 1 or more insomnia symptoms ≥ 3 times per week and a low incidence of help-seeking, reflecting an under-treatment of sleep disruption [34]. Along these lines, encouraging results have been associated with the behavioural treatment of sleep disorders, like insomnia, where cognitive behavioral approaches, in randomized controlled trials, demonstrate efficacy [35], raising the question of how such treatments could be applied to nocturnal hypertension. Caution must be taken, however, as the behavioural methods effectively applied in reducing blood pressure, diurnally, have not necessarily affected nocturnal pressures, suggesting specific tailoring for influencing autonomic arousal during sleep may be needed [36].

Although our study introduced novel findings, we must acknowledge limitations that indicate interpretive cautions. The sample was assessed while undergoing a Cardiac Rehabilitation Program, which bases referrals on recent cardiac events resulting in hospitalization. Accordingly, this group may represent a sample of patients more impaired with respect to cardiac function, compared to samples being followed by primary care physicians. Furthermore, the 24-h ambulatory monitoring assessment was voluntary and thus patients less able to tolerate ABPM were self-selected for exclusion. An additional point is that patients tolerate 24-h ABPM differently and thus some apparent nocturnal BP readings may be related to the variability of reactions to nighttime assessment. Until multiple serial ABPMs are undertaken, it is difficult to exclude the possibility that when patients better adjust to ambulatory assessments, their nocturnal BP reading may be reduced.

## Conclusions

In conclusion, our findings relate nocturnal hypertension (dipping vs. non-dipping) to fundamental indices of cardiac function and capacity but these factors accounted for a modest degree of variance. This suggests nocturnal BP is a complex multi-faceted phenomena, with mechanisms that may be better controlled through specific medical and behavioural treatments.

## Abbreviations

BP: Blood pressure
ANBP: Abnormal nocturnal blood pressure
ABPM: Ambulatory blood pressure monitoring
SBP: Systolic blood pressure
DBP: Diastolic blood pressure
HRR: Heart rate reserve
MAP: Mean arterial pressure
PP: Pulse pressure
HR: Heart rate
MET: Metabolic equivalent
MHR: Maximum heart rate
BMI: Body mass index
WC: Waist circumference
